# The Molecular Mechanisms of Intestinal Inflammation and Fibrosis in Crohn’s Disease

**DOI:** 10.3389/fphys.2022.845078

**Published:** 2022-02-11

**Authors:** Yuki Hayashi, Hiroshi Nakase

**Affiliations:** Department of Gastroenterology and Hepatology, Sapporo Medical University of Medicine, Sapporo, Japan

**Keywords:** intestinal fibrosis, IBD, Crohn’s disease, PPARγ, TLR4, AIEC, Th17, PAI-1

## Abstract

Crohn’s disease (CD) is an inflammatory bowel disease (IBD) with repeated remissions and relapses. As the disease progresses, fibrosis and narrowing of the intestine occur, leading to severe complications such as intestinal obstruction. Endoscopic balloon dilatation, surgical stricture plasty, and bowel resection have been performed to treat intestinal stenosis. The clinical issue is that some patients with CD have a recurrence of intestinal stenosis even after the medical treatments. On the other hand, there exist no established medical therapies to prevent stenosis. With the progressive intestinal inflammation, cytokines and growth factors, including transforming growth factor (TGF-β), stimulate intestinal myofibroblasts, contributing to fibrosis of the intestine, smooth muscle hypertrophy, and mesenteric fat hypertrophy. Therefore, chronically sustained inflammation has long been considered a cause of intestinal fibrosis and stenosis. Still, even after the advent of biologics and tighter control of inflammation, intestinal fibrosis’s surgical rate has not necessarily decreased. It is essential to elucidate the mechanisms involved in intestinal fibrosis in CD from a molecular biological level to overcome clinical issues. Recently, much attention has been paid to several key molecules of intestinal fibrosis: peroxisome proliferator-activating receptor gamma (PPARγ), toll-like receptor 4 (TLR4), adherent-invasive *Escherichia coli* (AIEC), Th17 immune response, and plasminogen activator inhibitor 1 (PAI-1). As a major problem in the treatment of CD, the pathophysiology of patients with CD is not the same and varies depending on each patient. It is necessary to integrate these key molecules for a better understanding of the mechanism of intestinal inflammation and fibrosis.

## Introduction

Crohn’s disease (CD) is a chronic inflammatory bowel disease (IBD) that progresses irreversibly, with more than 30% of the patients gradually developing intestinal fibrosis, which leads to complications, such as intestinal obstruction, perforation, and fistula (Rieder et al., 2013). The general mechanism of intestinal fibrosis is acute or chronic inflammation that leads to the destruction of the extracellular matrix (ECM) around the site of inflammation. Additionally, cytokines and growth factors, such as transforming growth factor (TGF-β), stimulate the ECM component cells, namely the intestinal myofibroblasts (Bettenworth and Rieder, 2017). This results in excessive ECM re-synthesis, which, in turn leads to intestinal fibrosis. In the case of CD, chronic inflammation is the main factor leading to intestinal fibrosis. The currently implemented treatments for intestinal stricture are limited to mechanical treatments, such as endoscopic balloon dilation, surgical strictureplasty, and bowel resection (Crespi et al., 2020). However, some patients with CD have recurrent intestinal stenosis, even after the mechanical treatment (Scarpa et al., 2003). Minimally invasive treatment strategies, specifically drug administration, are desirable for CD patients at risk of developing intestinal stenosis. Therefore, it is important to understand the underlying molecular mechanisms of inflammation and fibrosis for developing such therapies. In this review, we have attempted to provide a comprehensive description of the molecular mechanisms underlying intestinal inflammation and fibrosis in the case of CD.

## Pathological Characteristics of CD and Intestinal Stenosis

Crohn’s disease is a chronic IBD that mainly occurs in the small and large intestines. Histologically, it is characterized by the fibrosis-induced thickening of the intestinal wall, mainly the submucosal layer, and an increase in smooth muscle cell growth (Van Assche et al., 2004). The increased mRNA and protein expression of cytokines, such as TGF-β1 and insulin-like growth factor 1 (IGF-1), in all intestinal layers coincident with the inflammation sites and the increased deposition of ECM proteins synthesized by myofibroblasts induce intestinal fibrosis (Lawrance et al., 2001b; Fiocchi and Lund, 2011). Activated myofibroblasts are required to produce the ECM. These activated myofibroblasts include pre-existing myofibroblasts that are activated by inflammatory stimulants and de-differentiated mesenchymal cells [fibroblasts, smooth muscle cells, epithelial cells transformed by epithelial-mesenchymal transition (EMT), and endothelial cells transformed by endothelial-mesenchymal transition (EndoMT)]. There are patterns of differentiation of epithelial cells by EMT and endothelial cells by EndoMT, including stellate cells, pericytes, and bone marrow stem cells (Wynn, 2007). In the case of intestinal fibrosis caused by non-specific intestinal inflammation, once the inflammation subsides, the increased production of the fibrous matrix is suppressed, and the matrix metalloproteinase (MMP)-induced degradation of the fibrous matrix is promoted. Finally, intestinal fibrosis and the associated stenosis improve with a certain degree of plasticity (Rieder et al., 2007). However, in CD, even after the inflammation subsides, fibrosis progresses due to abnormal production of the fibrous matrix or reduced degradation of the matrix by the MMPs, both of which result in abnormal deposition of ECM (Lawrance et al., 2001a; Speca et al., 2012). This probably accounts for the high number of endoscopic or surgical treatments of stenosis in recent years, even after immunosuppressive therapies, such as biologics, have extensively improved the intestinal inflammation in CD (Rieder et al., 2017). Another significant feature of CD is thickened mesenteric fat (“creeping fat”; Schäffler and Herfarth, 2005). “Fat wrapping” is defined as the condition in which more than 50% of the intestinal surface is covered with adipose tissue, and the intestinal surface on the side of the foregut is also covered with fat (Sheehan et al., 1992). Creeping fat is predominantly found in CD patients, and it is generally absent in patients with ulcerative colitis (UC). The physiological implications of creeping fat have not yet been studied. However, recent reports have demonstrated that this massive fat (Desreumaux et al., 1999; Weisberg et al., 2003; Karmiris et al., 2006; Acedo et al., 2011).

## Peroxisome Proliferator-Activating Receptor Gamma

**Figure 1:**
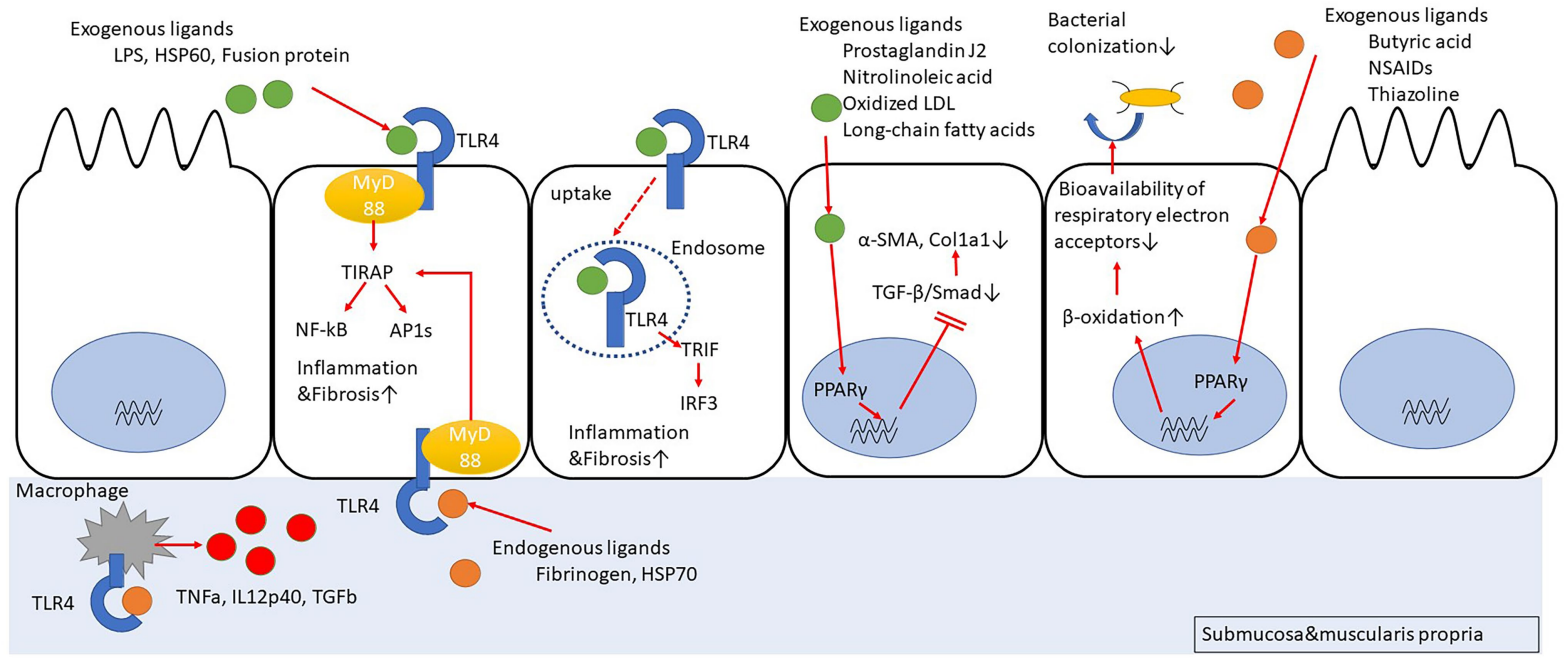
Peroxisome proliferator-activating receptor gamma (PPARγ) suppresses fibrosis by inhibiting Smad. PPARγ promotes cellular β-oxidation and inhibits bacterial colonization in intestinal epithelial cells. Toll-like receptor 4 (TLR4) stimulation induces inflammation/fibrosis. Macrophages activated with TLR4 stimulation produce pro-inflammatory cytokines.

Peroxisome proliferator-activated receptors (PPARs) are nuclear receptors that regulate the expression of genes involved in energy metabolism, cell development, and cell differentiation. There are three members of PPARs, namely PPARα, peroxisome proliferator-activating receptor gamma (PPARγ), and PPARβ/δ. Upon ligand binding, the PPARs translocate into the nucleus, form a heterodimer with retinoid X receptors, and bind to peroxisome proliferator-responsive elements (PPREs) to regulate the transcription of target genes (Mangelsdorf et al., 1995; Zhang et al., 2007; Chan and Wells, 2009; Figure 1). There have been reports about several ligands (full agonists, partial agonists, and antagonists) targeting the PPARs, based on which medical research and drug discovery have been actively pursued (Kroker and Bruning, 2015; Mirza et al., 2019). Incidentally, 5-aminosalicylic acid (5-ASA), which is widely used in the treatment of IBD, is also a PPARγ ligand (Rousseaux et al., 2005; Iacucci et al., 2010). The expression of PPARγ in the intestinal epithelium is possibly related to the intestinal microbiota composition. In fact, butyrate production by the intestinal microbiota activates PPARγ signaling in the colonic epithelium, thereby resulting in the β-oxidation of energy substrates in colonic epithelial cells (colonocytes). This, in turn, reduces the activity of respiratory electron receptors for intestinal bacteria, which may be pathogenic (Byndloss et al., 2017). Additionally, toll-like receptor 4 (TLR4)-transfected colonocytes have been proven to prevent the abnormal growth of potentially pathogenic bacteria. In TLR4-transfected cancer coli-2 (Caco-2) cells, the TLR4 signaling pathway upregulates PPARγ expression as well as the expression of a PPARγ-dependent reporter in an inhibitor of nuclear factor kappa-light-chain-enhancer of activated B cells (Iκβ)-dependent manner. Similarly, PPARγ expression is decreased in the colon of mice devoid of functional TLR4 (Lpsd/Lpsd mice; Yamamoto-Furusho et al., 2014). Mucosal biopsies from patients with active UC reveal a decreased expression of PPARγ mRNA, which is negatively correlated with the endoscopic severity of the disease (Dubuquoy et al., 2003; Yamamoto-Furusho et al., 2014). Although there have been no studies regarding the relationship between PPARγ expression and microbiota composition in the intestines of IBD patients, disruption of gut microbiota can result in inappropriate PPARγ signaling responses in the intestinal epithelial cells, leading to further growth of the pathogenic gut bacteria and contributing to the exacerbation of UC. Furthermore, PPARγ is associated with response to chemical stimuli. For instance, when mice with a targeted disruption of the PPARγ gene in the intestinal epithelial cells (generated using the villin-Cre transgene and floxed PPARγ allele) were treated with dextran sodium sulfate (DSS), the expressions of interleukin 6 (IL-6), IL-1β, and TGFα mRNAs were increased in their colons, as compared to the corresponding levels in the control mice (Adachi et al., 2006). Interestingly, in the DSS model mice, administration of pioglitazone or rosiglitazone, which are full agonists of PPARγ, can improve intestinal inflammation (Adachi et al., 2006; da Rocha et al., 2020). The novel 5-ASA analog, GED-0507-34 Levo (GED), is also able to activate PPARγ and suppress the expression of the primary protein markers of fibrosis, namely alpha-smooth muscle actin (α-SMA) and collagen I-II, by inhibiting the TGF-β/Smad pathway in the DSS mouse model as well as in human intestinal fibroblasts (Speca et al., 2016). Hence, PPARγ agonists can function as therapeutic targets that can cause suppression of inflammation and inhibition of inflammation-related fibrosis.

On the contrary, it is interesting to note that the PPARγ full agonists have the ability to induce differentiation of fibroblasts into adipocytes (Tontonoz et al., 1994a,b). Therefore, the use of PPARγ full agonists may induce submucosal fat deposition (SFD) due to adipocyte differentiation. The SFD is a condition in which a low-attenuation inner ring around the intestinal lumen is surrounded by a concentric, high-attenuation outer ring, known to radiologists as the “halo sign” on computed tomography (CT) imaging, and it has been implicated in the refractoriness of CD (Jones et al., 1986; Ahualli, 2007; Giaslakiotis et al., 2008). However, SFD is not necessarily specific for CD (Muldowney et al., 1995), and its correlation with creeping fat is unclear. Nevertheless, the adipocytes release pro-inflammatory and fibrotic cytokines; therefore, it would be desirable to use a partial agonist in PPARγ-targeted therapy to avoid inducing adipocyte differentiation.

To date, PPARγ-targeted therapy has been extensively researched for lifestyle-related diseases, such as diabetes and non-alcoholic fatty liver disease (NAFLD; Janani and Ranjitha Kumari, 2015; Cheng et al., 2019), but human applications have been partly undermined by metabolic dysregulation and carcinogenicity issues (Peters et al., 2012; Wright et al., 2014; Aghamohammadzadeh et al., 2015). Moreover, the mechanism of action of PPARγ agonists is complex; particularly, the mechanisms by which the ligands exhibit organ-specific sensitivity and strength as well as the differences in the temporal changes in response to ligands remain unclear. However, for the treatment of fibrosis in CD, the side effects may be improved by using localized and short- or medium-term drug administration instead of administering systemic and long-term drug therapy, as in the case of treating lifestyle-related diseases. In developing PPARγ-based treatment, the large number of PPARγ agonists that have already been discovered by computer and high-throughput screening proves to be advantageous (Lewis et al., 2010; Otake et al., 2011; Liu et al., 2015). Additionally, the newly-developed high-throughput screening of intestinal organoid models and data mining of previously screened PPAR ligands might accelerate the research and development (Rossi et al., 2018; Du et al., 2020; Lukonin et al., 2021; Rayner et al., 2021).

## Toll-Like Receptor 4

The TLR4 belongs to a family of single transmembrane receptor proteins called the TLRs that activate innate immune responses by recognizing bacterial and viral components. In mammals, 11 types of TLRs have been identified (Akira and Takeda, 2004; Figure 1). Initially, TLR4 was identified as a receptor for lipopolysaccharide (LPS) of Gram-negative bacilli (Hoshino et al., 1999). However, further studies have revealed that it also functions as a receptor for other exogenous factors, such as heat-shock protein 60 (HSP60) from fungi (Bulut et al., 2002), respiratory syncytial virus (RSV)-derived fusion protein (Kurt-Jones et al., 2000), and taxol from plants (Kawasaki et al., 2000), as well as host-derived endogenous factors (Okamura et al., 2001; Johnson et al., 2002), including fibrinogen (Smiley et al., 2001) and HSP70 (Vabulas et al., 2002). After TLR4 recognizes its ligands, the major downstream signaling pathways that are stimulated include activation of NF-κB and activator protein 1 (AP1) *via* myeloid differentiation primary response 88 (MyD88)-dependent toll/interleukin-1 receptor domain-containing adapter protein (TIRAP) or activation of interferon regulatory factor 3 (IRF3) *via* MyD88-independent toll/interleukin-1 receptor (TIR)-domain-containing adapter-inducing interferon-β (TRIF). The TLR4 signaling pathway can induce cytokine production, such as tumor necrosis factor-alpha (TNF-α) and type I interferon (IFN), B cell proliferation, and maturation of dendritic cells to activate infection defense mechanisms (Verstrepen et al., 2008; Watts et al., 2010; Kawasaki and Kawai, 2014). In a comparison of TLR expression in primary intestinal epithelial cells between healthy controls and IBD patients, it was revealed that in the healthy controls, TLR3 and TLR5 were predominantly expressed, with little expression of TLR2 and TLR4, whereas in the CD patients, TLR3 expression was predominantly decreased and TLR4 expression was increased (Cario and Podolsky, 2000; Brown et al., 2014). In fact, the number of macrophages strongly expressing TLR4 was high in the inflamed mucosal lamina propria of the colon in CD patients (Hausmann et al., 2002). Based on this observation, many studies focused on determining the association between CD and TLR4 polymorphism. However, the abovementioned significant association was observed only in European Caucasians (Arnott et al., 2004; Franchimont et al., 2004; Brand et al., 2005; Fries et al., 2005), but not in non-Caucasian individuals or in non-European countries (Oostenbrug et al., 2005; Zouiten-Mekki et al., 2009). Therefore, it is likely that there are regional differences regarding this association. A meta-analysis integrating the above studies revealed an association between TLR4 Asp 299 Gly and IBD susceptibility in Caucasians but not in Asians (Cheng et al., 2015). Additionally, the data suggested that the association of IBD susceptibility with TLR4 Thr 399 lle might only occur in Caucasians.

The relationship between TLR4 and colonic fibrosis has been investigated using TLR4 knockout (KO) mice with the DSS colitis model (Jun et al., 2020). The results indicate that the *TLR4* gene-deficient mice exhibit a reduced colonic inflammation as well as a decrease in the infiltration of macrophages into the colon, thereby resulting in reduced collagen deposition and intestinal fibrosis. Additionally, the production of TNF-α, IL-12p40, and TGF-β was reduced in the peritoneal macrophages of the mice lacking the *TLR4* gene.

Certain studies have demonstrated that the direct stimulation of TLR4 with LPS in myofibroblasts derived from mouse intestine might activate the myofibroblasts *via* multiple pathways, such as phosphoinositide 3 (PI3) kinase, p38 mitogen-activated protein kinase (MAPK), and NF-κB, ultimately contributing to innate immune responses (Otte et al., 2003; Walton et al., 2009). Furthermore, the accumulation of submucosal fibroblasts and collagen is reduced when MyD88-deficient mice are subjected to enteritis (Månsson et al., 2012; Zhao et al., 2020). However, the expressions of TLR2, TLR4, and TLR5 are much weaker in colonic myofibroblasts than in the crypt epithelial cells of IBD patients (Brown et al., 2014).

## Adherent Invasive *Escherichia coli*

**Figure 2:**
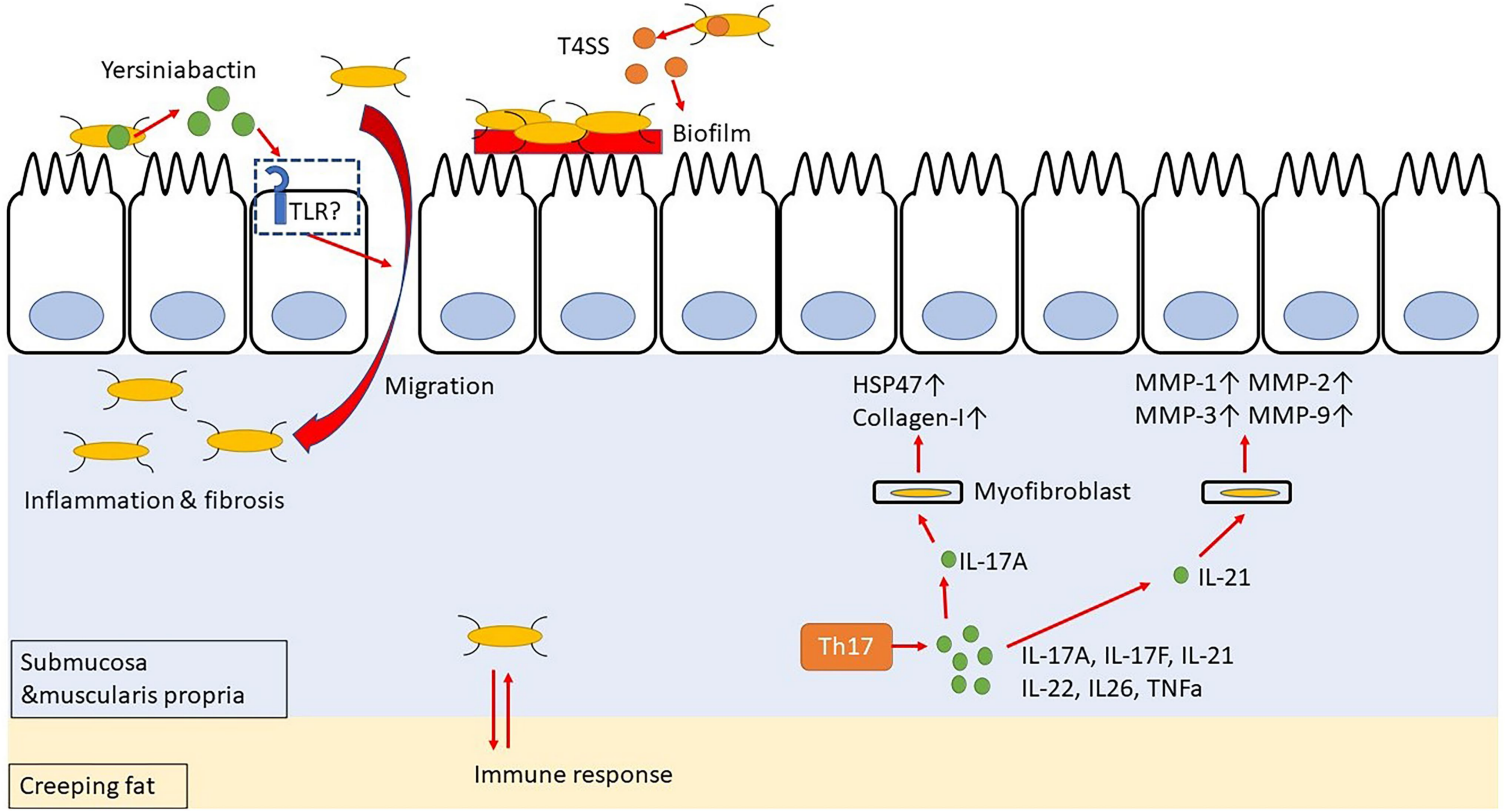
Yersiniabactin secreted by adherent-invasive *Escherichia coli* (AIEC) helps bacteria to transfer to submucosa, and bacteria in submucosa have mutual immune response with Creeping fat. Th17 cell-related cytokines promote fibrosis by acting on myofibroblast.

Incidentally, CD patients have an abnormal intestinal microflora composition, and these microorganisms are closely related to the inflammation and intestinal stenosis observed in CD. Specifically, a decreased occurrence of phylum Firmicutes and an increased occurrence of phylum Proteobacteria, especially Enterobacteriaceae, have been observed in CD patients with refractory inflammation or intestinal stricture (Frank et al., 2007; Sokol et al., 2020). Additionally, gene polymorphisms, as in the genes encoding autophagy-related 16 like 1 protein (*ATG16L1*) and nucleotide-binding oligomerization domain-containing protein 2 (*NOD2*; Hugot et al., 2001), are associated with the risk of developing CD (Hampe et al., 2007; Parkes et al., 2007). Adherent/invasive *Escherichia coli* (AIEC) in the intestine is most frequently isolated from the terminal part of the ileum of CD patients, thereby suggesting that AIEC may contribute to fibrosis (Darfeuille-Michaud et al., 2004; Small et al., 2013; Rieder et al., 2017). In fact, AIEC has been detected in 46.7% of CD patients compared to its occurrence in only 13.3% of healthy subjects (Sarabi Asiabar et al., 2018). AIEC requires a type IV secretion system (T4SS) to form biofilms in the intestinal tract and settle on the intestinal epithelial cells (Figure 2). Moreover, *Escherichia coli* isolates from CD patients are rich in T4SS, which is probably involved in the disease activity (Elhenawy et al., 2021). Interestingly, patients with serum antibodies to specific microbial peptides have an earlier onset of fibrostenosis and display early complications of CD (Dubinsky et al., 2008). Furthermore, creeping fat, specific for CD, promotes interaction with gut bacteria that have migrated into the submucosa, thereby contributing to the activation of immune responses (Suau et al., 2021). These data suggest that AIEC may exacerbate the inflammation and stenosis associated with CD. Additionally, AIEC secretes Yersiniabactin (Perry and Fetherston, 2011), an iron-chelating agent, to incorporate iron into its cells; however, this Yersiniabactin may help some bacteria to infect the subepithelial layers of the intestine, thereby causing inflammation and intestinal fibrosis (Kim et al., 2005; Ellermann et al., 2019).

## Th17 Cell Immune Response

The Th17 cells are differentiated from naïve T cells upon TGF-β and IL-6 stimulation (Sutton et al., 2006; Ruan et al., 2011), and they produce IL-17A, IL-17F, IL-21, IL-22, IL-26, and TNF-α (Cua and Tato, 2010; Hundorfean et al., 2012; Figure 2). The IL-17 stimulation activates the SEF/IL-17R (SEFIR) domain, which is closely related to the TIR domain of the IL-17R receptor. It thereby activates NF-κB and AP-1 signaling through NF-κB activator 1 (ACT-1) and tumor necrosis factor receptor (TNFR)-associated factor 6 (TRAF-6), respectively (Moseley et al., 2003; Gaffen, 2009; Wang et al., 2013). The NF-κB and AP-1 signaling induce the secretion of IL-1, IL-6, TNF-α, MMPs, and antimicrobial peptides. Hence, IL-17 has a pro-inflammatory role and can protect against extracellular parasitic as well as bacterial infections (Ye et al., 2001; Raffatellu et al., 2008; Lin et al., 2009). Incidentally, IL-17 and IL-21 are overexpressed in the colonic mucosa of UC patients, while IL-17, IL-21, and IL-22 are overexpressed in the colonic mucosa of CD patients (Fujino et al., 2003; Andoh et al., 2005; Monteleone et al., 2005; Brand et al., 2006). Therefore, the Th17-related cytokines are involved in the pathophysiology of both UC and CD. In CD, IL-17+ CD3+ T cells and CD68+ cells are scattered in the submucosa and muscularis propria, and some of these cells produce IFN-γ (Fujino et al., 2003; Annunziato et al., 2007). Genome-wide association studies have revealed that IL23R and five genes involved in Th17 differentiation, namely the *IL12B*, Janus kinase 2 (*JAK2*), signal transducer and activator of transcription 3 (*STAT3*), C-C motif chemokine receptor 6 (*CCR6*), and *TNFF15*, are associated with the susceptibility to CD (Barrett et al., 2008).

In patients with CD, IL-17A is overexpressed in the stenotic intestine, as compared to its expression in the tissues of the non-stenotic area (Biancheri et al., 2013). Interestingly, both IL-17A and HSP47 expressions are enhanced in the colons of patients with active CD. Moreover, IL-17A promotes the expression of HSP47 and collagen I in intestinal myofibroblasts and CCD-18Co cells isolated from patients. In fact, knockdown of HSP47 in these cells inhibits IL-17A-induced collagen I production (Honzawa et al., 2014). Additionally, IL-17A treatment of IEC-6 cells (a rat small intestinal cell) induces EMT, decreases E-cadherin expression, and increases vimentin, snail, and α-SMA expression (Zhang et al., 2018). It has also been reported that IL-21 boosts the Th1 response, which, in turn, stimulates the intestinal fibroblasts to secrete MMPs in response of CD (Monteleone et al., 2006). Incidentally, both Th1 and Th17 immune responses are involved in the trinitrobenzene sulfonic acid (TNBS)-induced colitis mice model (Zhu et al., 2012). In fact, administration of an anti-IL-17 antibody to mice with chronic colitis, which was induced by repeated intra-rectal administration of TNBS, decreases the expression of fibrosis-related cytokines, such as collagen 3, TNF-α, TIMP metallopeptidase inhibitor 1 (TIMP-1), and MMP-2, as well as inflammatory cytokines, namely IL-1β, TGF-β1, and TNF-α, ultimately resulting in a suppressed fibrosis (Zhang et al., 2018; Li et al., 2020). However, anti-IL-17A antibody (secukinumab) and anti-IL-17-receptor antibody (brodalumab) failed to demonstrate efficacy in the treatment of CD (Hueber et al., 2012; Mozaffari et al., 2015). Therefore, rather than direct inhibition of IL-17, IL-17 downstream pathways, such as HSP47, and other IL-17-based cytokines, such as IL-21, may be targets for avoiding intestinal fibrosis.

## Plasminogen Activator Inhibitor-1

Plasminogen activator inhibitor-1 (PAI-1) is a protein with a molecular weight of approximately 42,700 Da. It is mainly synthesized and secreted by vascular endothelial cells and hepatocytes. However, adipocytes and certain other cells also contribute to its production (Sillen and Declerck, 2021). Incidentally, PAI-1 is an inhibitor that regulates the fibrinolytic reaction by precisely forming a 1:1 irreversible bond with tissue plasminogen activator (tPA) and thereby inactivating it. Clinically, blood PAI-1 levels help to understand the pathogenesis of disseminated intravascular coagulation (DIC), a disease of the coagulation-fibrinolysis system (Gando et al., 2016; Hoshino et al., 2020; Morrow et al., 2021). Additionally, the expression of PAI-1 increases with age (Yamamoto et al., 2005). Elevated levels of TNF-α, IL-6, and TGF-β induce the expression of PAI-1 (Samad et al., 1999; Alessi et al., 2000; Rega et al., 2005). Subsequently, PAI-1 suppresses tPA production and prevents the conversion of plasminogen into plasmin, thereby resulting in a decrease in MMPs and the consequent inhibition of tissue fiber degradation (Munakata et al., 2015). Incidentally, PAI-1 is a major downstream target of TGF-β signaling, and its transcription is directly regulated by Smad3 (Samarakoon and Higgins, 2008). In IBD patients as well as in mice colitis models, the expression of PAI-1 is extensive inactive lesions, and PAI-1 and its direct target tPA play an essential role in the regulation of intestinal inflammation (Alkim et al., 2011; Kaiko et al., 2019; Su et al., 2020). Moreover, in the intestinal mucosa of the terminal ileum of patients with active CD, TGF-β and PAI-1 levels are elevated with a positive correlation (Imai et al., 2020). Mice with TNBS-induced intestinal fibrosis also exhibit elevated PAI-1, and administration of TM5275, which blocks PAI-1/tPA complex formation, in these mice leads to an increase in MMP9 expression that can ameliorate fibrosis (Ibrahim et al., 2014; Yahata et al., 2017; Imai et al., 2020).

## Conclusion and Prospects

In recent years, there has been an expansion in the knowledge regarding the associations between organ fibrosis and the underlying molecular pathways or functions. This may help to elucidate the molecular mechanism of intestinal fibrosis with respect to IBD. However, large-scale screening of the molecular structure, toxicity, and therapeutic efficacy of the potential therapeutic agents is essential. Hence, further development and improvement of high-throughput screening techniques, such as computer screening, organoid-based screening, and nematode-based screening (de Sousa Figueiredo et al., 2021) are desirable for the development of novel treatment strategies for CD.

## Author Contributions

YH drafted the manuscript. YH and HN critically revised the manuscript. All authors contributed to the article and approved the submitted version.

## Funding

This work was supported in part by Health and Labor Sciences Research Grants for research on intractable diseases from the MHLW of Japan (Investigation and Research for intractable Inflammatory Bowel Disease to HN) and JSPS KAKENHI Grant Number JP 21K07919 (to YH). The funders of the study had no role in the study design, data collection, data analysis, data interpretation, or writing of the report.

## Conflict of Interest

The authors declare that the research was conducted in the absence of any commercial or financial relationships that could be construed as a potential conflict of interest.

## Publisher’s Note

All claims expressed in this article are solely those of the authors and do not necessarily represent those of their affiliated organizations, or those of the publisher, the editors and the reviewers. Any product that may be evaluated in this article, or claim that may be made by its manufacturer, is not guaranteed or endorsed by the publisher.
